# Investigating the Role of Cerebral Amyloid Angiopathy in the Development of Amyloid‐Related Imaging Abnormalities

**DOI:** 10.1002/alz70861_108664

**Published:** 2025-12-23

**Authors:** Anouk R Geiser, Brianna R Colletti, Praveen Bathini, Cynthia A Lemere

**Affiliations:** ^1^ Ann Romney Center for Neurologic Diseases, Brigham and Women's Hospital, Boston, MA USA; ^2^ Maastricht University, Maastricht Netherlands; ^3^ Harvard Medical School, Boston, MA USA; ^4^ Brigham and Women's Hospital, Harvard Medical School, Boston, MA USA

## Abstract

**Background:**

Anti‐amyloid immunotherapy is associated with vascular side effects known as amyloid‐related imaging abnormalities (ARIA). This side effect potentially occurs in association with cerebral amyloid angiopathy (CAA). Sasmita et al. (Neuron, 2025) demonstrated that paternal inheritance of the 5xFAD gene led to twice the amount of amyloid plaque burden compared to maternal inheritance. Therefore, we investigated whether parental lineage and associated differences in amyloid burden influence the induction of microhemorrhages in anti‐amyloid immunized 5XE4 mice in this pilot study.

**Method:**

5xFAD mice (line 6799) available from Jackson Laboratory were crossed with floxed human APOE4 knock‐in mice provided by Cure Alzheimer’s Fund. This study included 17 mice: five with paternal and 12 with maternal inheritance of the 5xFAD gene. Nine 5XE4 mice (6 from the maternal 5xFAD lineage and 3 from the paternal lineage) were immunized with 20mg/kg of the 3D6 anti‐amyloid monoclonal antibody, and eight (6 from the maternal 5xFAD lineage and 2 from the paternal lineage) received an IgG2a isotype control. Injections were administered intraperitoneally weekly over seven weeks between five and seven months of age. Total amyloid burden, CAA, microhemorrhages, and CAA‐associated microhemorrhages in brain were analyzed by immunohistochemical and histological staining methods and quantified using Image J.

**Result:**

Paternal inheritance of the 5xFAD gene led to significantly more fibrillar amyloid (*p* =0.0153), including CAA (*p* =0.0073), than maternal inheritance. Seven weeks of anti‐amyloid immunotherapy resulted in a non‐significant 39% reduction of fibrillar amyloid compared to IgG2a‐treated control mice. 3D6 treatment additionally induced significantly more overall hemosiderin staining (*p* =0.0131) and CAA‐associated microhemorrhages (*p* =0.0155) compared to treatment with IgG2a. Comparing microhemorrhages between mice of paternal and maternal lineage, we found that 3D6‐immunized mice of the paternal lineage had double the amount of microhemorrhages compared to 3D6‐immunized mice from the maternal lineage (ns).

**Conclusion:**

Mice with higher levels of vascular amyloid (paternal lineage) developed more microhemorrhages upon anti‐amyloid immunization with 3D6 than mice with less vascular amyloid (maternal lineage). This strong association for higher incidence of microhemorrhages with increased vascular amyloid confirms the important role for CAA in ARIA. Funding: NIH NINDS 1R01NS136122 and Cure Alzheimer’s Fund (CAL)

